# βKlotho Suppresses Tumor Growth in Hepatocellular Carcinoma by Regulating Akt/GSK-3β/Cyclin D1 Signaling Pathway

**DOI:** 10.1371/journal.pone.0055615

**Published:** 2013-01-30

**Authors:** Xiaoming Ye, Yu Guo, Qi Zhang, Wenjie Chen, Xuefeng Hua, Wei Liu, Yang Yang, Guihua Chen

**Affiliations:** 1 Department of General Surgery, Lingnan Hospital, Sun Yat-sen University, Guangzhou, Guangdong, China; 2 Guangdong Provincial Key Laboratory of Liver Disease Research, Guangzhou, Guangdong, China; 3 Hepatology Laboratory, Hospital for Liver Disease, Sun Yat-Sen University, Guangzhou, Guangdong, China; Medical University Graz, Austria

## Abstract

βKlotho is a regulator in multiple metabolic processes, while its role in cancer remains unclear. We found the expression of βKlotho was down-regulated in human hepatocellular carcinoma tissues compared with that in paired adjacent non-tumourous liver tissues. Hepatoma cells also showed decreased expression of βKlotho compared with normal hepatocyte cells. Reintroduction of βKlotho into hepatoma cells inhibited their proliferation. The anti-proliferative effect of βKlotho might be linked with G1 to S phase arrest, which was mediated by Akt/GSK-3β/cyclin D1 signaling, since forced expression βKlotho reduced the phosphorylation level of Akt and GSK-3β and induced down-regulation of cyclin D1. Furthermore, βKlotho overexpression could inhibit tumorgenesis, while constitutively activated Akt could override the suppressive effects of βKlotho *in vivo*. These data suggest βKlotho suppresses tumor growth in hepatocellular carcinoma.

## Introduction

βKlotho is a single-pass transmembrane protein belonging to the Klotho family. The extracellular domain of βKlotho consists of two internal repeats (βKL1 and βKL2) sharing homology with members of the family 1 glycosidases but lacking glucosidase catalytic activity [1]. βKlotho is predominantly expressed in the liver, pancreas and white adipose tissue[1]. The function of βKlotho was unknown until Ito and colleagues showed that βKlotho-null mice exhibited increased synthesis and excretion of bile acid by elevating mRNA levels of CYP7A1 and CYP8B1, two important enzymes in the bile acid biosynthetic pathway[2]. Previous studies have demonstrated that βKlotho is involved in the control of bile acid and lipid and glucose metabolism in liver and adipocytes[2], [3]. Recently, it was reported that βKlotho could also inhibit proliferation of tumor cells [4]. However, another study showed βKlotho had an oncogenic role[5]. Therefore, the exact role of βKlotho in tumorigenesis is still unclear.

βKlotho usually forms a complex with fibroblast growth factor (FGF) receptors and functions as a co-receptor for FGFs, especially the FGF19 subfamily members, which consist of FGF15 (the mouse ortholog of human FGF19), FGF21, and FGF23[6], [7]. Of the four FGF receptors (FGFR), FGFR4 is dominant in mature hepatocytes[8]. The presence of βKlotho confers high affinity binding of FGFs to FGFR4 and results in activation of ERK1/2 signaling and depression of Akt signaling[4].

Hepatocellular carcinoma (HCC) is the fifth most common cancer and the third leading cause of cancer-related mortality in the world[9], [10]. However, the molecular mechanism of HCC is still poorly understood. The cell cycle is a critical regulator of the processes of cell proliferation. Uncontrolled cell proliferation is the hallmark of cancer, and tumor cells typically acquire damaged genes that directly regulate the cell cycle[11]–[13]. cyclin D1 is one of the more frequently altered cell cycle regulators in cancers. Deregulated function of cyclin D1, often resulting from overexpression of the protein, has been documented in numerous human cancers, including HCC[14]–[18]. cyclin D1 regulates the G1 to S phase transition of the cell cycle by binding to Cdk4 or Cdk6 and by phosphorylating pRb[13]. The cyclin D1 expression level is mediated by Akt/GSK-3β signaling. Akt phosphorylates and inactivates GSK-3β resulting in stabilization of cyclin D1[19]–[21]. GSK-3β could inhibit cyclin D1 gene transcription by inaction of its transcription factor β-catenin. On the other side, GSK-3β could also induce cyclin D1 proteolysis by direct phosphorylation of cyclin D1. Overall, inactivation of GSK-3β and subsequent up-regulation of cyclin D1 have a critical role in cell cycle and HCC.

In the present study, we examined the role of βKlotho in hepatocarcinogenesis. Our data showed that βKlotho expression was frequently decreased in primary HCC tissues and was also significantly down-regulated in HCC cell lines. Furthermore, overexpression of βKlotho into hepatoma cells inhibited their proliferation. The anti-proliferative effect of βKlotho might be linked with G1to S phase arrest, which was mediated by the Akt/GSK-3β/cyclin D1 pathway. Finally, reintroduction of βKlotho could suppress tumorigenesis in the xenograft mouse model and this effects could be aborted by Akt activity. These findings suggest βKlotho suppresses tumor growth in HCC.

## Materials and Methods

### Ethics statement

The study was approval from the Institutional Research Ethics Committees of the third affiliated hospital of Sun Yat-sen university, and written informed consent was obtained from all patients. All animal procedures in this study were approved by the Animal Experimentation Ethics Committee of Lingnan Hospital, Sun Yat-sen University.

### Tissues Samples

Samples of tumor and adjacent non-tumorous liver tissues were obtained from patients who had undergone primary HCC curative hepatic resection at the third affiliated hospital of Sun Yat-sen university, Guangzhou, China. Immediately after resection, all tissues were snap-frozen in liquid nitrogen and stored at -80°C.

### Cell lines, Constructs and Transfection

Human hepatocyte cells (L02) and human hepatoma cell lines (HepG2, Hep3B) were cultured as reported [22]. The other two human hepatoma cell lines, SMMC-7721 and Huh 7, were reported previously [23]. The human βKlotho gene was cloned from L02 cells and using the following primers: forward, 5′-AATTGCGGCCGCATGAAGCCAGGCTGTGC-3′; reverse, 5′-AATTGGATCCTTAGCTAACAACTCTCTTGCCTT-3′. The resulting βKlotho PCR product was digested with NotI and BamHI and ligated into p3×FLAG-CMV-7.1 expression vector (Sigma-Aldrich, St. Louis, MO) to obtain the βKlotho expression vector. Constitutively activated myristoylated-Akt (myr-Akt) cDNA expression vector was purchased from Upstate (Charlottesville, VA). All transfections used Lipofectamine 2000 (Invitrogen, Carlsbad, CA) according to the manufacturer’s protocol.

### Immunohistochemistry (IHC)

The slides were deparaffinized through xylenes and graded ethyl alcohols and then rinsed in water, followed by quenching of endogenous peroxidase activity by a 0.3% solution of hydrogen peroxidase in methanol for 30 min. Antigen retrieval was performed by microwave-heating in sodium citrate buffer (10 mM, pH 6.0). Sections were blocked with 1% normal serum in PBS for 1h and then incubated with anti-βKlotho antibody (Abcam, Cambridge, MA) overnight at 4°C. Bound anti-body was detected by the avidin-biotin-peroxidase complex method, using the Elite ABC kit (Vector Laboratories, Burlingame, CA) as recommended by the manufacturer.

IHC staining was quantitatively analysed using Axio-Vision computerized image analysis system assisted with the automatic measurement program (Carl Zeiss Jena Gmbh, Jena Germany). Briefly, the stained sections were evaluated at 200× magnification and ten representative staining fields of each section were analysed to verify the mean optical density (MOD), which represented the strength of staining signals as measured per positive pixel. All the experiments were performed independently three times at least.

### Western Blotting

Cells or tissues were lysed for total protein extraction in radio-immunoprecipitation assay (RIPA) buffer (50 mM Tris-HCl, pH 7.4, 150 mM NaCl, 1% NP-40, 0.25% Na-deoxycholate, 1 mM EDTA, 1 mM NaF) together with a protease inhibitor cocktail (Sigma-Aldrich, St. Louis, MO). Proteins were separated by 10% SDS-poly-acrylamide gel electrophoresis and transferred to poly-vinylidene difluoride membranes (Amersham Life Science, Piscataway, NJ). Membranes were incubated for an hour in a blocking buffer containing 5% nonfat dry milk and then probed with antibodies against βKlotho (Abcam, Cambridge, MA), phospho-Akt Ser473, total Akt, phospho-GSK-3β Ser9, total GSK-3β, total cyclin D1(Cell Signaling Technology, Inc, Danvers, MA) and tubulin (Sigma-Aldrich, St. Louis, MO) as indicated. The bound primary antibodies were then probed with respective secondary antibodies labeled with horseradish peroxidase. Immunolabeled proteins were detected by using the ECL system (Amersham Life Science, Piscataway, NJ). Band intensities were quantified using NIH ImageJ software. All the experiments were performed independently three times at least.

### Colony Formation Assay

Cells were transfected with either vector or βKlotho. Two days following transfection, the Hep3B or SMMC-7721 cells were stripped and plated on 6-well culture dishes, and G418 (500 µg/ml, Sigma-Aldrich, St. Louis, MO) was added to the culture media to select the transfected cells. Every 3 days the medium was replaced with fresh medium containing G418. Colonies were stained using crystal violet and counted 2 weeks after transfection. All the experiments were performed in triplicate wells three times.

### MTT Viability Assay

The viability of the cells was assessed by 3-(4,5-dimethylthiazol-2-yl)-2,5-diphenyltetrazolium bromide (MTT, Sigma-Aldrich, St. Louis, MO) assay. A total of 1×10^3^ cells per well were plated in 96-well with triplicate wells for each transfection, and incubated for 24 h in 100 µl culture media. Cells were transfected with either vector or βKlotho. MTT (500 mg/ml) was added to the cells and cultivated for another 4 h. After the medium was aspirated, the cells were dissolved by dimethyl sulfoxide (Sigma-Aldrich, St. Louis, MO). Absorbance of the formazan product was measured by an enzyme-linked immunosorbent assay reader. Each assay was repeated three times.

### Flow Cytometric Analysis

The effect of βKlotho on cell cycle was checked in Hep3B or SMMC-7721 cells by propidium iodide staining and flow cytometry. Briefly, 1×10^6^ cells were harvested at 48h after transfection, washed in PBS and fixed in ice cold 70% ethanol for 1 hour. RNA was digested by incubating the samples with 1 mg/ml RNase A (Invitrogen, Carlsbad, CA) for 30 min at 37°C. Propidium iodide (50 µg/ml, Sigma-Aldrich, St. Louis, MO) was then added and the samples were recorded using the Navios Flow Cytometers (Beckman Coulter, Miami, FL). Cell cycle analysis was performed with the use of Multi Cycle for Windows (Phoenix Flow Systems, San Diego, Calif.). Experiments were repeated in triplicate. Average values and standard deviation statistical analyses were computed.

### *In Vivo* Tumorigenesis Assay

Hep3B or SMMC-7721cells (5×10^6^ cells suspended in 100 µl PBS) transfected with vector, βKlotho or βKlotho plus myr-Akt were injected subcutaneously into the dorsal left flank of 4-week-old male Balb/c nude mice. Tumor diameter was measured every 2–3 days for 4 weeks. Tumor volume (mm^3^) was estimated by measuring the longest and shortest diameter of the tumor and calculated using the following formula: volume  =  0.5×(shortest diameter)^2^×(longest diameter)[23]. Mice were sacrificed and the tumor weights were measured. All the experiments were performed independently three times at least.

### Statistical Analysis

Data were presented as the mean ± SD error of the mean. Student’s t test was used for comparison among different groups. The correlation of βKlotho expression with various clinicopathologic parameters were calculated with χ^2^ test. The difference in tumor growth rate between the two groups of nude mice was determined by repeated-measures analysis of variance. *p* < 0.05 was considered statistically significant.

## Results

### Decreased expression of βKlotho in HCC

**Figure 1:**
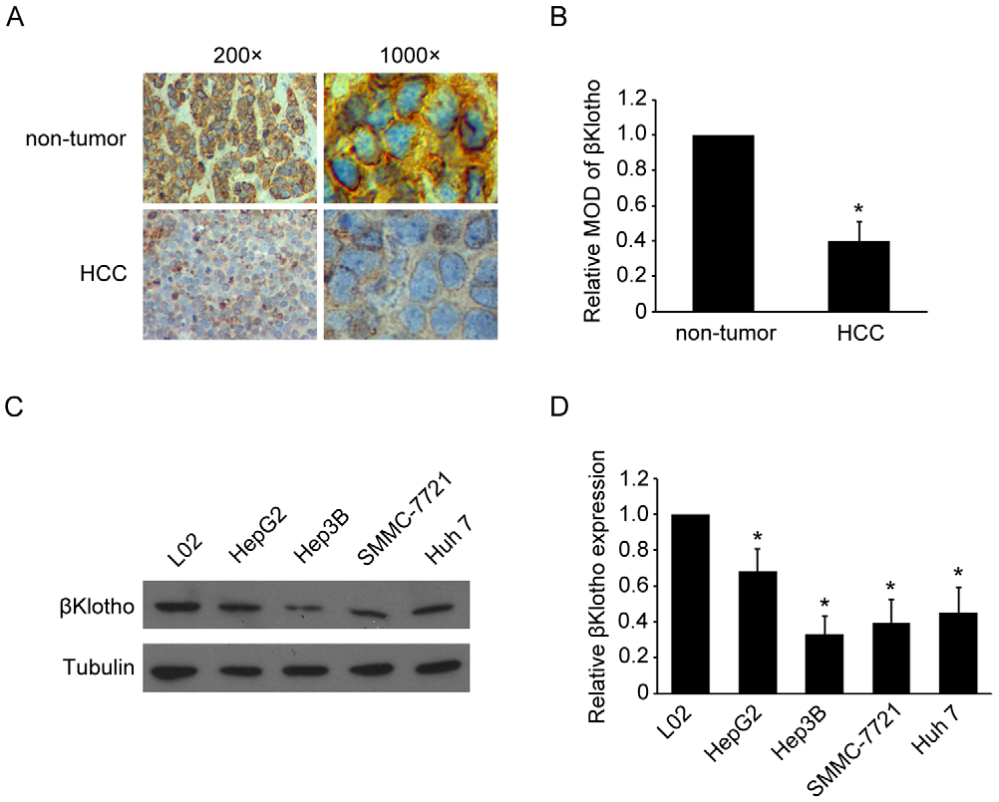
Decreased expression of βKlotho in HCC tissue and hepatoma cell lines. (A) Immunohistochemical analysis of βKlotho protein expression in non-tumor liver samples and HCC samples. Representative photographs were taken at ×200 or ×1000 magnifications. (B) Statistical quantification of relative MOD of βKlotho staining in non-tumor liver samples and HCC samples (47 cases). (C) Western blot analysis and (D) statistical quantification of βKlotho expression in hepatoma cell lines (HepG2, Hep3B, SMMC-7721 and Huh 7) and normal hepatocyte cell line (L02). Tubulin expression levels were used as internal controls. * indicates *p* < 0.05. The experiments were performed independently three times at least.

To study the role of βKlotho in HCC, we first examined the expression pattern of βKlotho in 47 paired HCC samples and adjacent non-tumor tissue samples obtained from the same patients. Immunohistochemistry analysis revealed that βKlotho expressed abundantly in non-tumor tissue samples, while was less detectable in HCC samples (Fig. 1A, Table 1). Quantitative analysis indicated that the mean optical density (MOD) of βKlotho staining in HCC tissue samples were statistically significantly lower than the value in adjacent non-tumor tissue samples (Fig. 1B). The βKlotho expression in hepatoma cell lines (HepG2, Hep3B, SMMC-7721 and Huh 7) and normal hepatocyte cell line (L02) were also analyzed by western blotting. Compared with L02, the expression of βKlotho reduced in all the hepatoma cell lines (Fig. 1C, 1D). These data showed decreased expression of βKlotho in HCC tissue and hepatoma cell lines.

**Table 1:** Relationship between βKlotho expression and clinicopathologic features of patients with hepatocellular carcinoma.

Features	High βKlotho expression	Low βKlotho expression	p value
Mean age (years)	63.5	61.9	
Gender			0.56
Male	9	24	
Female	5	9	
Tumor Size (cm)			0.59
< 2	8	15	
≥ 2	6	16	
Differentiation			0.86
Well	3	7	
Moderate	7	14	
Poor	4	12	
Liver cirrhosis			0.37
Yes	10	19	
No	4	14	
Metastasis			0.32
Yes	9	16	
No	5	17	
HBsAg status			0.41
Positive	11	22	
Negative	3	11	
Serum AFP			0.61
Positive	10	21	
Negative	4	12	

### βKlotho overexpression inhibits HCC cell proliferation

**Figure 2:**
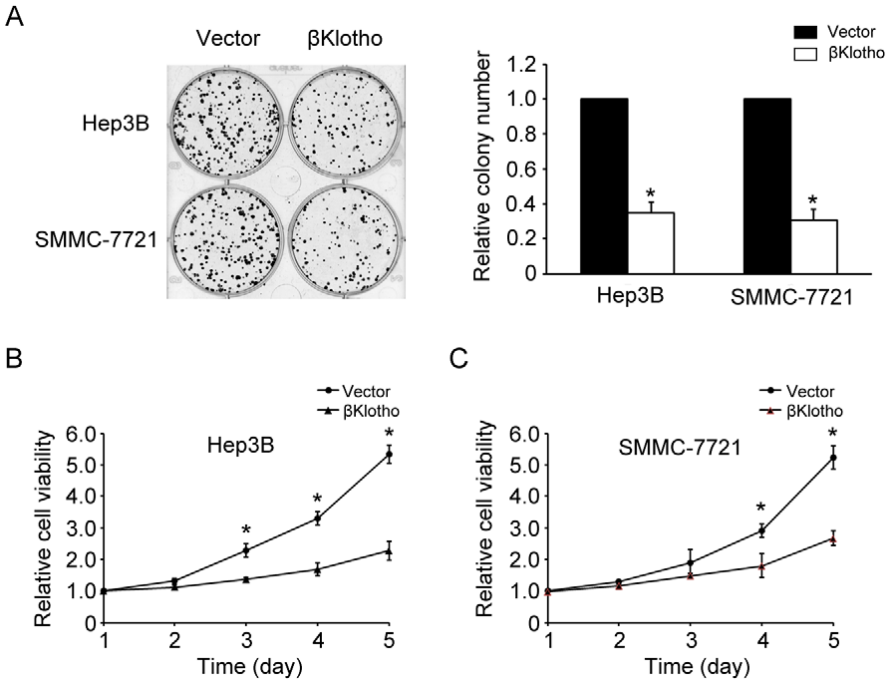
βKlotho overexpression inhibited hepatoma cell proliferation. (A) Colony formation assay. Representative micrographs (left panel) and quantification (right panel) of crystal violet-stained Hep3B or SMMC-7721 cells transfected with either vector or βKlotho. (B) Viability of βKlotho-transfected or vector-transfected Hep3B cells were determined by MTT assay on days 1 to 5 after transfection. (C) Viability of βKlotho-transfected or vector-transfected SMMC-7721 cells were determined by MTT assay on days 1 to 5 after transfection. Each bar represents the average ± SD of three independent experiments. * indicates *p* < 0.05.

Since there was an inverse correlation between the expression of βKlotho and HCC, we further explored the functional role of βKlotho in HCC progression. The effect of βKlotho on growth was assessed by colony formation assay. Hep3B or SMMC-7721 cells were transfected with either vector or βKlotho. We found that the colony numbers of βKlotho-transfected cells were substantially decreased compared with cells transfected with vector alone (Fig. 2A). We also examined the effect of varying levels of βKlotho expression on cell growth. Hep3B cells were transfected with 0, 0.1, 1.0 or 5.0 ug βKlotho plasmids. The colony formation assay showed the inhibitory effect of βKloth on hepatoma cell growth was in a dose-dependent manner (Fig. S1A). Such a proliferation inhibitory activity of βKlotho was further demonstrated by the MTT viability assay. Reduction of viability was observed in βKlotho-transfected cells (Fig. 2B and 2C). To exclude the possibility that these effects resulted from a non-βKlotho mutation, another βKlotho-transfected cell clone was used and exhibited similar effects in colony formation assay and MTT viability assay (Fig. S1B, S1C and S1D). Collectively, these data suggest that βKlotho has an anti-proliferation role in hepatoma cells.

### βKlotho overexpression induces G1 to S phase arrest of hepatoma cells, in association with cyclin D1 down-regulation

**Figure 3:**
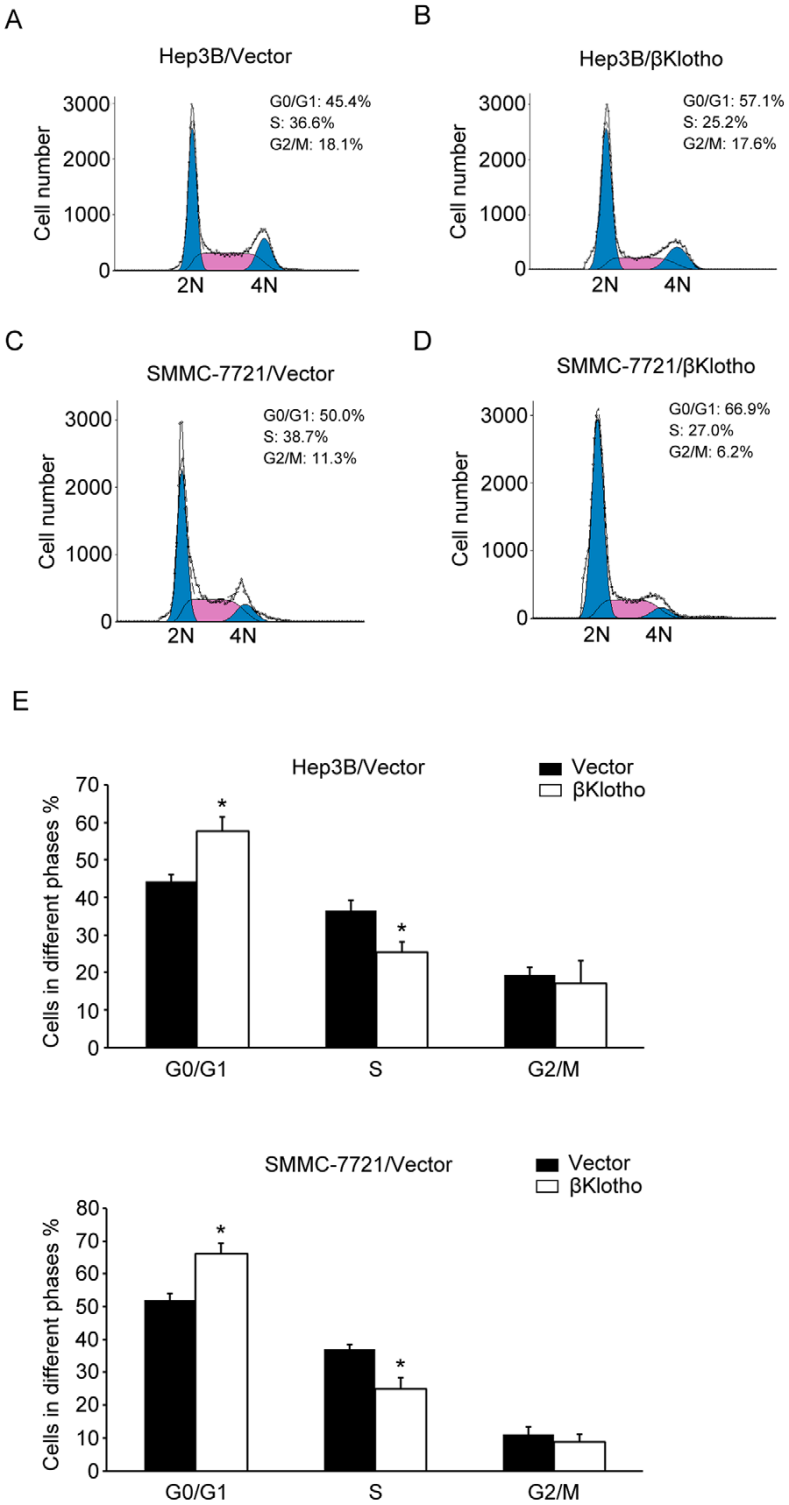
βKlotho overexpression induced G1 to S phase arrest of hepatoma cells. (A, B) A representative data of flow cytometric analysis of Hep3B cells transfected with vector or βKlotho. (C, D) A representative data of flow cytometric analysis of SMMC-7721 cells transfected with vector or βKlotho. (E) The cell percentages in G0/G1, S and G2/M phase were measured in three independent experiments. * indicates *p* < 0.05.

**Figure 4:**
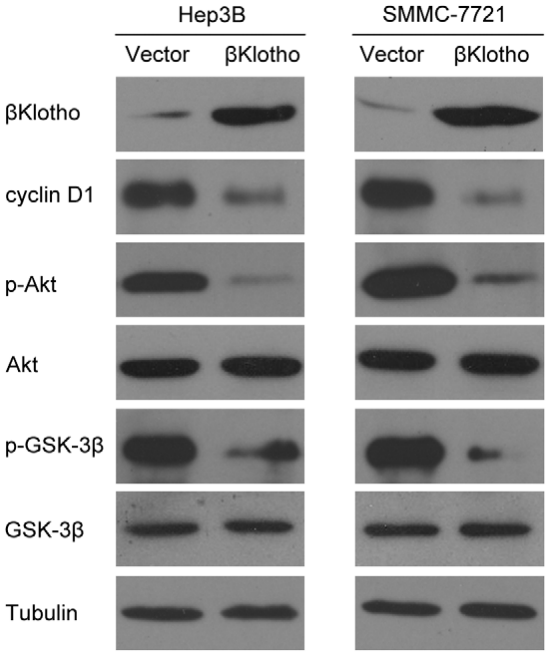
Regulation of Akt/GSK-3β/cyclin D1 signaling pathway by βKlotho. Western blotting analysis of βKlotho, cyclin D1, phosphorylated Akt (p-Akt), Akt, phosphorylated GSK-3β (p-GSK-3β), GSK-3β and tubulin levels in the indicated hepatoma cell lines transfected with vector or βKlotho. The experiments were performed independently three times at least.

To investigate the mechanism that mediated the anti-proliferation function of βKlotho, flow cytometry analysis was performed. Overexpression of βKlotho increased the percentage of cells in G0/G1 peak but decreased that in S peak (Fig. 3), indicating that βKlotho induce G1 to S phase arrest of hepatoma cells. Accordingly, we tested the expression of cyclin D1, a critical regulator of the transition from G1 to S phase in cell cycle. Western blotting analysis revealed that βKlotho overexpressed successfully and the expression of cyclin D1 was dramatically down-regulated in βKlotho-transfected cells (Fig. 4). Furthermore, we examined the Akt/GSK-3β signaling, which plays a critical role in cyclin D1 expression and carcinogenesis. Forced expression βKlotho reduced the phosphorylation level of Akt and GSK-3β (Fig. 4), indicating an increased activity of GSK-3β. Another βKlotho-transfected cell clone also exhibited similar effects (Fig. S2). These data suggested the anti-proliferative effect of βKlotho is associated with cyclin D1 degradation induced G1to S phase arrest.

### Overexpression of βKlotho Suppresses Tumor Formation

**Figure 5:**
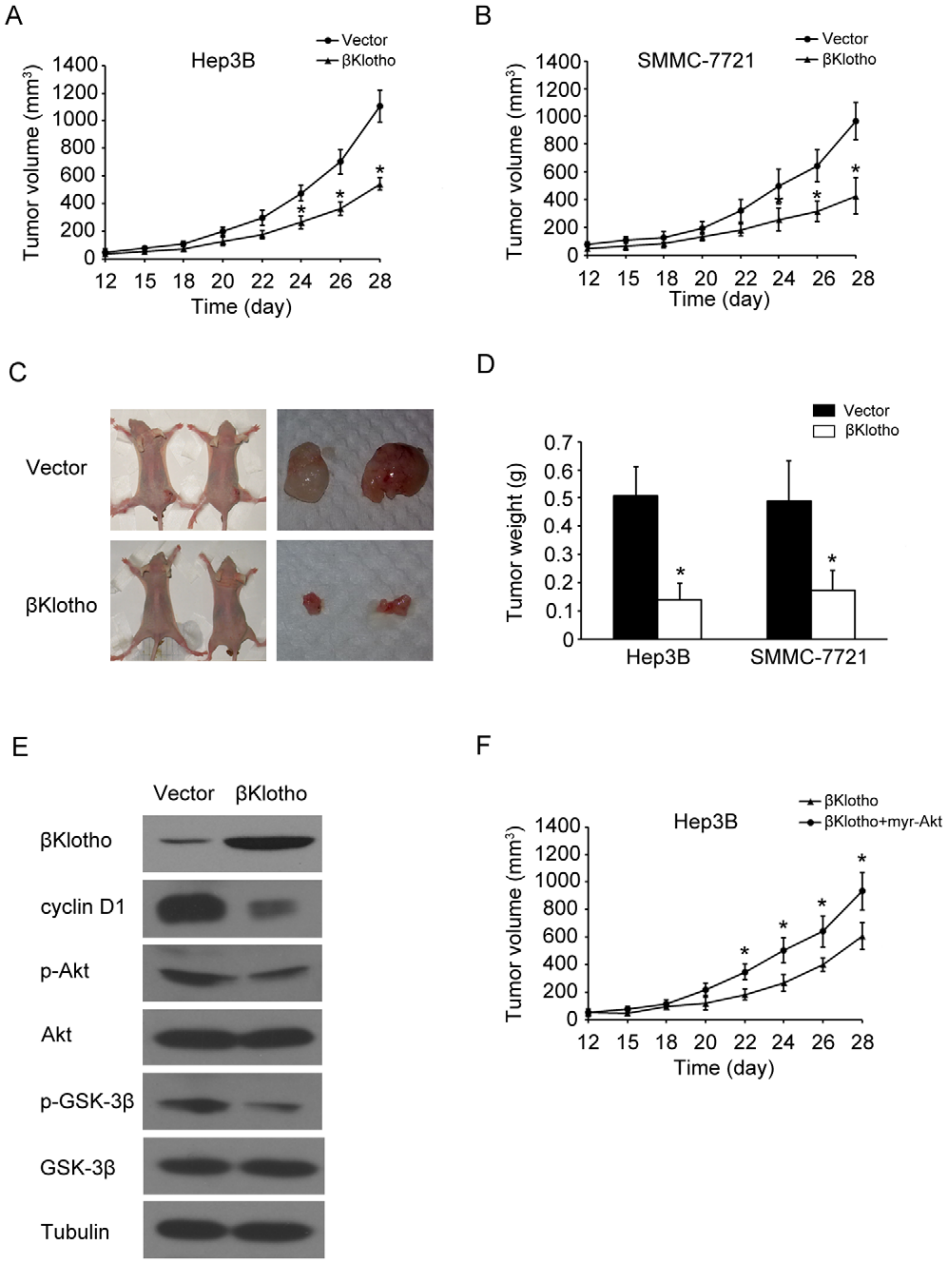
Overexpression of βKlotho suppressed tumor formation. (A, B) Subcutaneous tumor growth curve of βKlotho-transfected Hep3B or SMMC-7721 cells in nude mice was compared with vector transfected cells. The βKlotho group showed a retarded tumor growth compared to the vector group. (C) A representative picture of tumor growth in nude mice subcutaneously inoculated with vector or βKlotho transfected hepatoma cells. The βKlotho group showed a retarded tumor growth compared to the vector group. (D) The mean tumor weights in nude mice subcutaneously inoculated with vector or βKlotho transfected hepatoma cells. (E) Western blotting analysis of βKlotho, cyclin D1, phosphorylated Akt (p-Akt), Akt, phosphorylated GSK-3β (p-GSK-3β), GSK-3β and tubulin levels in the subcutaneous Hep3B cells tumor samples. (F) Subcutaneous tumor growth curve of βKlotho-transfected Hep3B cells in nude mice was compared with βKlotho and myr-Akt co-transfected cells. The βKlotho plus myr-Akt group showed a retarded tumor growth compared to the βKlotho alone group. The data were means ± SD of three separate experiments. * indicates *p* < 0.05.

To determine whether βKlotho was involved in tumorgenesis, we further examined the effect of βKlotho on tumorgenesis *in vivo* using a xenograft mouse model. Hepatoma cells were transfected with vector or βKlotho, and then injected subcutaneously into nude mice to initiate tumor formation. At 4 weeks after tumor cell inoculation, large tumors were seen in the vector groups, while the tumor volume was still minimal in those mice transplanted with the βKlotho-expression cells (Fig. 5A, 5B). At the end of experiments tumors were isolated (Fig. 5C) and the mean tumor weight was significantly less in βKlotho-transfected nude mice as compared with the vector control mice (Fig. 5D). These results were consistent with the anti-proliferation function of βKlotho and indicated that βKlotho overexpression elicited a strong anti-tumor effect on HCC *in vivo*. We also analyzed the Akt/GSK-3β/cyclin D1 signaling in these tumors. We confirmed that βKlotho was overexpressed successfully. βKlotho-transfected tumors showed a decreased expression level of cyclin D1 and phosphorylation level of Akt and GSK-3β (Fig. 5E), which were similar to the results *in vitro*. To demonstrate the βKlotho effects in suppressing tumor xenograft growth was occurring through Akt/GSK-3β/cyclin D1 signaling pathway, hepatoma cells were co-transfected with βKlotho and constitutively activated Akt (myr-Akt). The Akt activity could override the suppressive effects of βKlotho (Fig. 5F). Taken together, we found that overexpression of βKlotho suppressed tumor formation by regulating Akt/GSK-3β/cyclin D1 signaling pathway.

## Discussion

Our observations identified βKlotho could suppress tumor growth in HCC. We found that βKlotho expression was frequently decreased in primary HCC tissues compared with their adjacent non-tumor tissues, and was also significantly down-regulated in hepatoma cell lines. Furthermore, reintroduction of βKlotho into hepatoma cells inhibited their proliferation. The anti-proliferation effect of βKlotho might be linked with G1to S phase arrest, which was mediated by the Akt/GSK-3β/cyclin D1 signaling. βKlotho overexpression could also suppress tumorigenesis in the xenograft mouse model, while constitutively activated Akt could override the suppressive effects of βKlotho. These findings suggest βKlotho has an anti-tumorigenic role in HCC.

βKlotho is a metabolic regulator and is involved in bile acid biosynthesis[2], [24]. The βKlotho-null mice exhibit increased synthesis and excretion of bile acid. It is reported that chronically higher levels of bile acids can promote liver tumor formation, suggesting an intriguing link between metabolic regulation and HCC[25], [26]. Recently, βKlotho was found down-regulated in hepatoma cells and could inhibit tumor cell proliferation[4]. However, another study demonstrated that βKlotho was elevated in HCC tissues and βKlotho-silencing decreased cell proliferation[5]. These results are conflicting and thus the exact role of βKlotho in hepatocarcinogenesis has remained unclear. We and others found that βKlotho is predominantly expressed in normal liver tissue[1], while its expression was frequently decreased in primary HCC tissues and hepatoma cell lines. This implied βKlotho had a blocking effect on HCC. Moreover, reintroduction of βKlotho into hepatoma cells inhibited their proliferation. Besides the *in vitro* data, we also revealed that βKlotho could also reduce tumor genesis ability *in vivo*. These results demonstrated βKlotho has an anti-tumorigenic role in HCC. Moreover, βKlotho interacts with FGFR4 to form a complex and the βKlotho-FGFR4 partnership mediates some biological functions[4]. Several studies showed that FGFR4 played no positive role in liver regeneration and limited hepatocarcinogenesis using FGFR4 knockout mice, suggesting a negative role of FGFR4 in tumorigenesis[27], [28]. These data are consistent with the conclusion that βKlotho could suppress tumor growth.

Cell cycle governs the transition from quiescence to cell proliferation, and is typically divided into four phases. The periods associated with DNA synthesis (S phase) and mitosis (M phase) are separated by gaps of varying length called G1 and G2 phase. The majority of human cancers have been reported to have alterations in the function of cell cycle regulatory proteins[11]–[13]. cyclin D1 is one of the key regulatory proteins controlling the transition from G1 to S phase. We found that βKlotho could induce cell cycle arrest at the G1 to S phase transition, in association with down-regulation of cyclin D1. Given that disruption of the regulatory system controlling G1 phase progression is a common event in human hepatocarcinogenesis and cyclin D1 overexpression plays a carcinogenic role in HCC[29], our data suggested βKlotho inhibited hepatoma cells growth by down-regulation of cyclin D1.

βKlotho acts as a co-receptor and facilitates metabolic signaling by FGFs. The βKlotho-FGFR4 partnership causes a depression of Akt signaling[4]. Consistent with this, we showed that βKlotho overexpression reduced the phosphorylation of Akt and subsequent phosphorylation of GSK-3β, indicating Akt inactivation and GSK-3β activation respectively. This might contribute to cyclin D1 degradation because GSK-3β is a critical regulator of cyclin D1 expression[19]–[21]. Moreover, the Akt/GSK-3β signaling also plays an important role in HCC[30]–[32]. Thus, our data suggested the Akt/GSK-3β/cyclin D1 signaling pathway mediated the function of βKlotho in hepatoma cells proliferation and hepatocarcinogenesis.

In summary, we identified that βKlotho could suppress tumor growth in HCC, and our investigation suggested that restoration of βKlotho would be a potential molecular target for HCC therapy.

## Supporting Information

Figure S1 **βKlotho overexpression inhibited hepatoma cell proliferation.** (A) βKloth inhibited hepatoma cell growth in a dose-dependent manner. Hep3B cells were transfected with 0, 0.1, 1.0 or 5.0 ug βKlotho plasmids. The expression levels were confirmed by Western blotting. Crystal violet-stained cells were quantified. (B) Quantification of crystal violet-stained Hep3B or SMMC-7721 cells transfected with another clone of βKlotho in colony formation assay. (C, D) The viability of Hep3B cells and SMMC-7721 cells transfected with another clone of βKlotho was determined by MTT assay on days 1 to 5 after transfection. Each bar represents the average ± SD of three independent experiments. * indicates *p* < 0.05.
(TIF)

Figure S2 **Regulation of Akt/GSK-3β/cyclin D1 signaling pathway by another clone of βKlotho.** Western blotting analysis of βKlotho, cyclin D1, phosphorylated Akt (p-Akt), Akt, phosphorylated GSK-3β (p-GSK-3β), GSK-3β and tubulin levels in the indicated hepatoma cells transfected with vector or another clone of βKlotho. The experiments were performed independently three times at least.
(TIF)
