# Supplementary figures and images for: βKlotho Suppresses Tumor Growth in Hepatocellular Carcinoma by Regulating Akt/GSK-3β/Cyclin D1 Signaling Pathway
Source: PLoS One. 2013 Jan 30;8(1):e55615. doi: 10.1371/journal.pone.0055615 (PMC3559476; doi:10.1371/journal.pone.0055615)

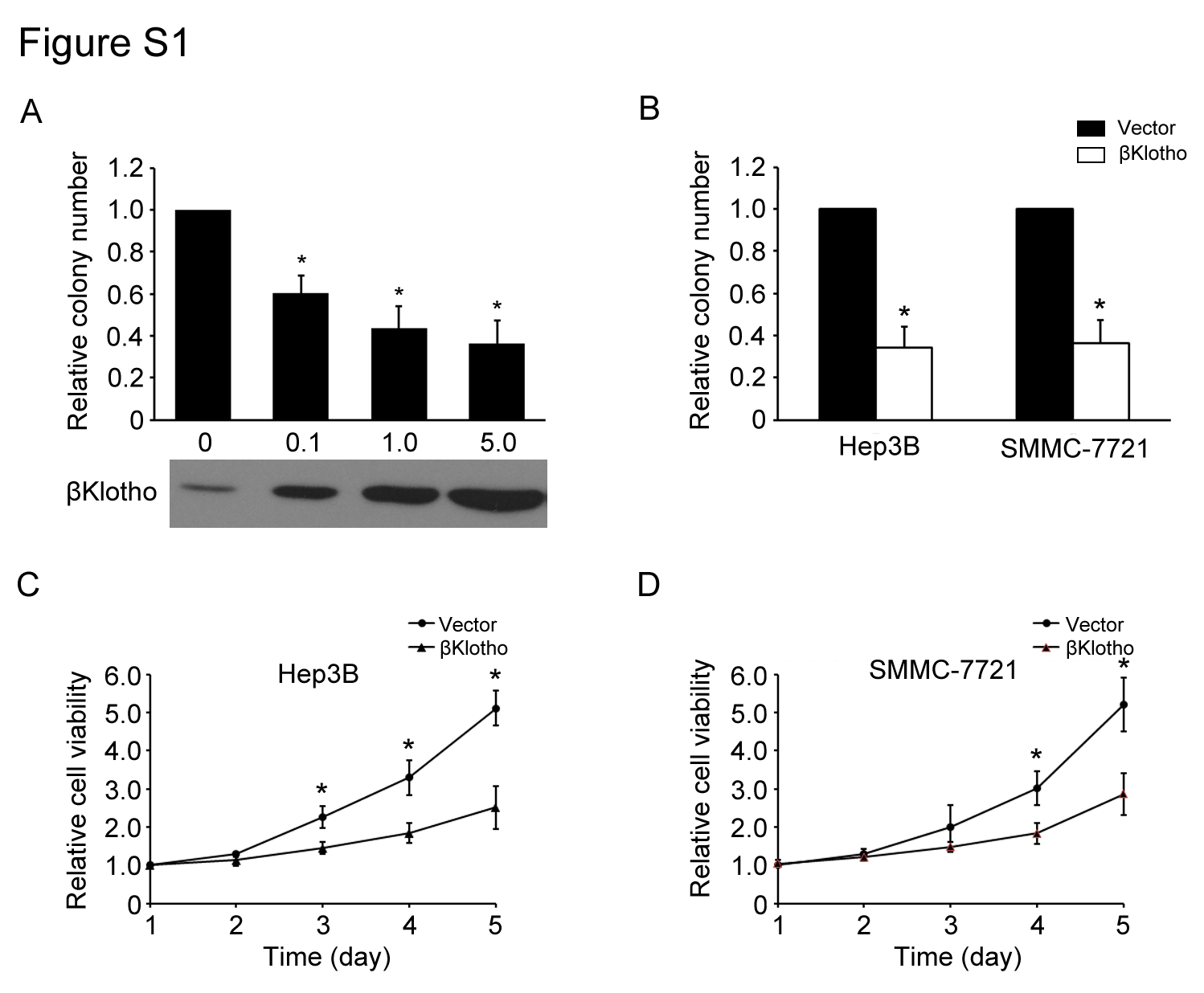

Supplement: Figure S1 — βKlotho overexpression inhibited hepatoma cell proliferation. (A) βKloth inhibited hepatoma cell growth in a dose-dependent manner. Hep3B cells were transfected with 0, 0.1, 1.0 or 5.0 ug βKlotho plasmids. The expression levels were confirmed by Western blotting. Crystal violet-stained cells were quantified. (B) Quantification of crystal violet-stained Hep3B or SMMC-7721 cells transfected with another clone of βKlotho in colony formation assay. (C, D) The viability of Hep3B cells and SMMC-7721 cells transfected with another clone of βKlotho was determined by MTT assay on days 1 to 5 after transfection. Each bar represents the average ± SD of three independent experiments. * indicates p < 0.05. (TIF) [file pone.0055615.s001.tif]

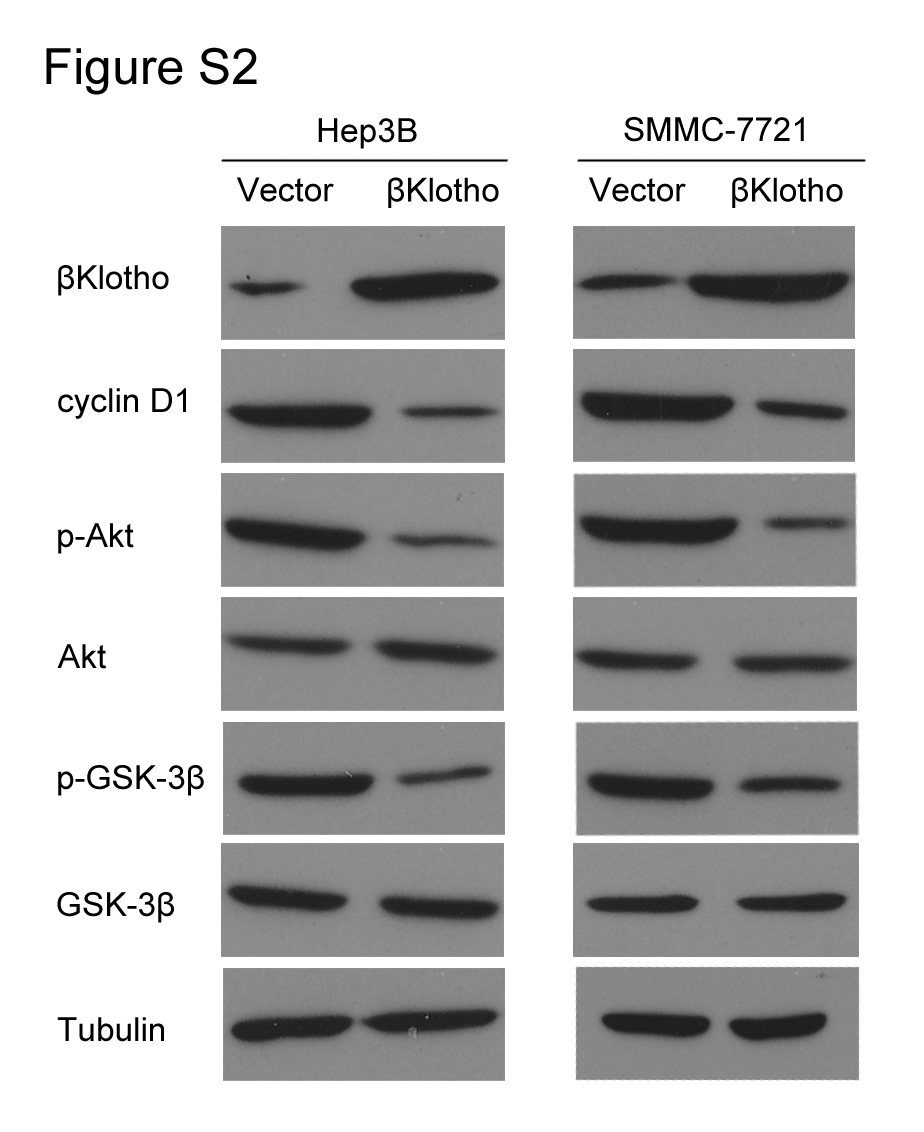

Supplement: Figure S2 — Regulation of Akt/GSK-3β/cyclin D1 signaling pathway by another clone of βKlotho. Western blotting analysis of βKlotho, cyclin D1, phosphorylated Akt (p-Akt), Akt, phosphorylated GSK-3β (p-GSK-3β), GSK-3β and tubulin levels in the indicated hepatoma cells transfected with vector or another clone of βKlotho. The experiments were performed independently three times at least. (TIF) [file pone.0055615.s002.tif]
